# Unbiased Confidence Intervals for Psychological Testing: The Regression-Based True Score Approach With Scale Correction

**DOI:** 10.1177/10731911251362532

**Published:** 2025-09-04

**Authors:** Stefan C. Schmukle

**Affiliations:** 1 Leipzig University, Germany

**Keywords:** confidence interval, psychological testing, psychological assessment, classical test theory

## Abstract

Two different approaches for calculating confidence intervals (CIs) for individual scores in psychological testing practice have been discussed in the literature within the framework of classical test theory. The traditional approach (CI: observed score ± *z* · standard error of measurement) has been criticized because it does not consider the phenomenon that, with imperfect measurement, true scores will be closer to the population average than the observed scores (regression to the mean). The regression approach (CI: regression-based true score estimate ± *z* · standard error of the estimate) takes this effect into account, but has the disadvantage that it leads to confidence intervals that are on a different scale than the observed scores. The different scaling occurs because true scores have a smaller standard deviation than observed scores, and the extent of this shrinkage depends on the reliability of the test. Here, I suggest a scale correction for the regression-based true score estimate to preserve the original scaling. Simulations indicate that this approach has the desired properties and outperforms the two existing approaches. The regression approach with scale correction is therefore recommended for calculating confidence intervals for individual scores in psychological testing practice.

When interpreting the result of a psychological test, it is important that test administrators do not take a participant’s observed score at face value. Rather, they need to also consider measurement error that occurs due to imperfect reliability. For this purpose, a confidence interval (CI) has to be computed, which includes the true score with a given probability (in practice, 95% CIs are commonly used), around the participant’s observed score. Psychologists are trained to calculate these confidence intervals in their curricula, and all textbooks on psychological assessment cover this topic (e.g., Anastasi, 1976; Cohen et al., 2013; Crocker & Algina, 2006; Finch & French, 2019; Murphy & Davidshofer, 2005; Nunnally & Bernstein, 1994).

The calculation of confidence intervals can be based on either classical test theory (CTT) or item response theory (IRT), depending on which theoretical framework was used to construct the psychological test being administered. Although the CTT makes more or less unrealistic assumptions of linearity and equivalence of indicators, leading, for example, to the questionable expectation that the size of the confidence intervals is invariant across participants with different true scores, most psychological tests used in clinical practice are still based on CTT. For this reason, it is important to know how to calculate confidence intervals within the CTT framework as accurately as possible, despite the known weaknesses of the CTT.

However, even though the estimation of confidence intervals based on CTT is considered basic textbook knowledge, the literature in fact describes several approaches (for an overview, see Charter & Feldt, 2001) that supposedly result in accurate and unbiased confidence intervals. Whereas some of these approaches seem more like idiosyncratic suggestions by individual authors and lack a clear theoretical conceptualization (Charter & Feldt, 2001), two of these approaches have actually been discussed more frequently in the literature and have also found their way into textbooks (Crocker & Algina, 2006; Finch & French, 2019; Nunnally & Bernstein, 1994; Sijtsma, & Van der Ark, 2021): the traditional approach and the regression-based true score approach.

## The Traditional Approach

The *traditional approach* for estimating a confidence interval is based on the fact that in CTT, the observed score *x_j_* of a test examinee *j* is the sum of the true score *t_j_* and an error score *e_j_*. The expected range (ER) of the observed score *x_j_* can then be estimated using the standard error of measurement (SEM), which depends on the reliability *r_xx_* and the standard deviation of the observed score *s_x_*:

(1) $$\mathrm{SEM}=\;s_{x}\;\cdot \;\sqrt{1-r_{\mathrm{xx}}}$$

The ER of the observed score for a test participant with true score *t_j_* is then given by:

(2) $$\mathrm{ER}=t_{j}\pm z\;\cdot \;\mathrm{SEM}$$

where *z* is the unit normal value for the desired confidence level (e.g., *z* = 1.96 for the usual 95% CI).

In the traditional approach, the confidence interval for the true score given an observed score is then estimated analogously to the ER. Whereas the ER is centered on the true score, the confidence interval for the true score given an observed score is estimated with its center on the observed score:

(3) $$\mathrm{CI}=x_{j}\pm z\;\cdot \;\mathrm{SEM}$$

This equation is regularly taught in psychology curricula and is also presented in most textbooks on psychological testing (e.g., Anastasi, 1976; Cohen et al., 2013; Crocker & Algina, 2006; Murphy & Davidshofer, 2005). It has also been shown that the observed score is a maximum-likelihood estimate of the true score, assuming continuous and normally distributed test scores (Mellenbergh, 1996), providing some justification for the traditional approach.

## The Regression Approach

Nevertheless, it has long been pointed out that the traditional approach leads to biased estimates. Such criticism can be found not only in the psychometric literature (Dudek, 1979; Lord & Novick, 1968; McHugh, 1957; Nunnally & Bernstein, 1994) but also in the literature on clinical psychology (Brophy, 1986; Knight, 1983). The problem is that the participant’s true score tends to be closer to the population mean than the observed score. This effect is called regression to the mean, and is one factor that, for example, causes post-therapy effects to regress toward the group mean (Hsu, 1995). This effect gets stronger the more the observed result deviates from the mean and the lower the reliability of the test.

Technically, this regression to the mean is caused by the fact that observed scores are necessarily correlated with error scores under the assumptions of CTT: Observed scores are defined as the sum of true scores and error scores (*x_j_* = *t_j_* + *e_j_*), and thus, given that true scores and error scores are uncorrelated, observed scores are necessarily correlated not only with true scores but also with error scores. The correlation between observed and error scores is given by the following equation:

(4) $$r_{\mathrm{xe}}=\sqrt{1-r_{\mathrm{xx}}}$$

Because observed scores and error scores are positively correlated, and error scores by definition have a mean of zero, participants with above-average observed scores are more likely to have positive error scores, and participants with below-average observed scores are more likely to have negative error scores. Therefore, compared to the observed scores, the actual true scores tend to be closer to the mean of the population from which the participant was drawn, and even more so the lower the reliability.

For this reason, the confidence interval for the true score should not be centered on the observed score as in Equation (3) but on the regression-based estimated true score (ETS; formula first introduced by Kelley, 1927):

(5) $$\mathrm{ETS}=\;M_{x}+r_{\mathrm{xx}}(x_{j}-M_{x})$$

The regression-based ETS is a weighted sum of the participant’s observed score *x_j_* and the average score *M_x_* on that test in the norming sample (e.g., *M_x_* = 50 in case of *T*-scores). In the extreme case of a reliability of one, the ETS is equal to the observed score, whereas for a reliability of zero, the ETS is always identical to the group mean, regardless of the test score. In the latter case, any deviation of the test score from the mean is just measurement error.

Importantly, the standard error of the regression-based ETS, abbreviated as the standard error of the estimate (SEE), is not equal to the SEM but is given by the following equation (a derivation of Equations (5) and (6) based on the assumptions of CTT can be found on p. 43 in Gulliksen, 1950; for a derivation using the Bayesian framework, see Levy & Mislevy, 2016, pp. 155–159):

(6) $$\mathrm{SEE}=\;s_{x}\;\cdot \;\sqrt{r_{\mathrm{xx}}}\;\cdot \;\sqrt{1-r_{\mathrm{xx}}}$$

The confidence interval for the *ETS approach* (also called the regression-based approach) can then be estimated by:

(7) CI=ETS±z·SEE

The regression-based true score approach has repeatedly been recommended over the traditional approach as more accurate (Brophy, 1986; Dudek, 1979; Lord & Novick, 1968; McHugh, 1957; Nunnally & Bernstein, 1994). Nevertheless, the regression approach has not prevailed in test practice, as is evident from the title of Dudek’s article, “the continuing misinterpretation of the standard error of measurement.” One reason the traditional approach might be preferred is that the regression approach is computationally more complicated than the traditional approach. To address this issue, confidence intervals based on estimated true scores rather than observed scores have been published for various tests (Atkinson, 1991; Knight, 1983; Kramer, 1993), and nowadays more and more tests report confidence intervals for estimated true scores rather than observed scores in their manuals.

## Problems With the Regression Approach

**Figure 1:**
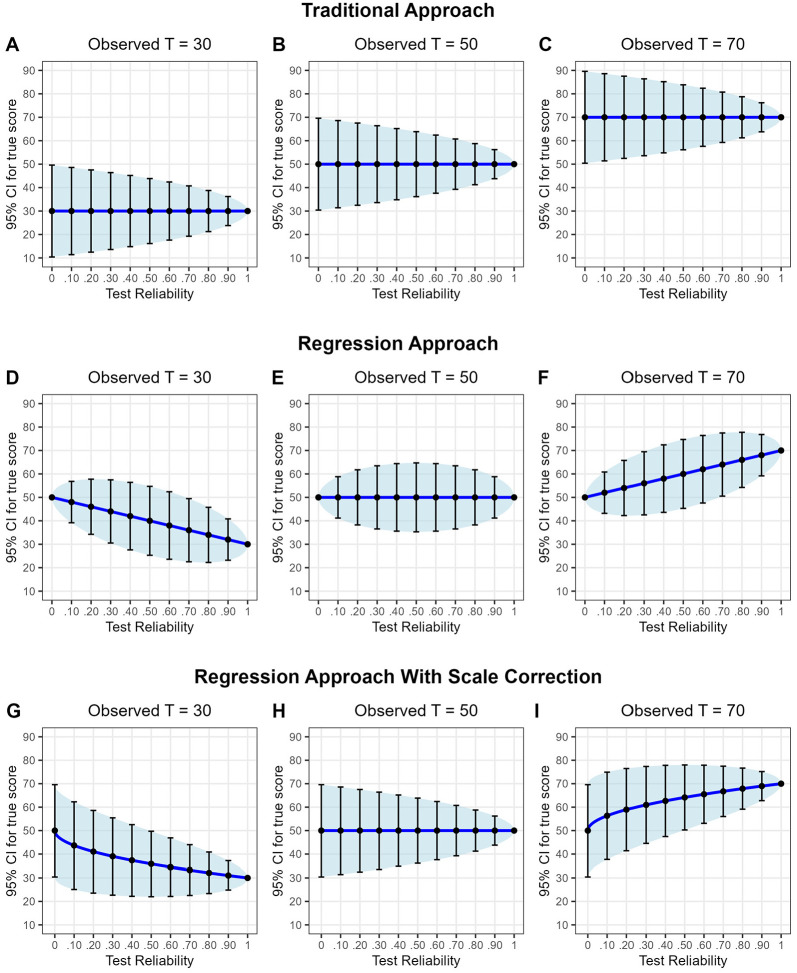
Confidence intervals (95% CIs) for the true score using the three different statistical approaches depending on the size of the observed *T*-score (*M* = 50, *SD* = 10) and test reliability.

Another reason why the traditional approach is often still preferred over the regression approach, however, may be that the confidence intervals calculated with the regression approach have some anomalies. To illustrate this point, Figure 1 shows confidence intervals for participants with observed *T*-scores (defined as *M* = 50 and *SD* = 10) of 30, 50, and 70 plotted against the test reliability. Panels A to C in the first row show the confidence intervals calculated with the traditional approach, and Panels D to F in the middle row show the confidence intervals for the regression approach. We see two main differences.

First, the regression approach considers regression to the mean, that is, participants’ estimated true scores are closer to the mean than their observed scores (see Figure 1, Panels D and F). This effect is stronger the greater the deviations of the observed values from the mean and the lower the reliability. This particular behavior of the approach makes sense from a diagnostic point of view because it integrates a priori information about the distribution of the true test scores.

Second, whereas for the traditional approach, the width of the confidence intervals consistently decreases with increasing reliability, confidence intervals behave quite differently in the regression approach. Here, for reliabilities below .50, confidence intervals become wider as reliability increases. Afterward, with increasing reliability above .50, they shrink again. This tendency is a consequence of Equation (6), which has its maximum at *r_xx_* = .50; but from a diagnostic point of view, it is puzzling. Why should it be possible that in some scenarios (reliability below .50), with increasing reliability of the information, our certainty decreases?

Panel E illustrates this unexpected result using a participant with an observed *T*-score of 50. For this participant, the regression-based ETS is always 50 regardless of the test’s reliability, but once the reliability falls below .50, the lower the reliability, the narrower the confidence interval around the ETS of 50. This tendency is obviously nonsensical from a diagnostic point of view because measurement error increases steadily as reliability decreases, so that the confidence interval should become wider rather than narrower. For a reliability of zero, the confidence interval even has a width of zero (i.e., the participant seems to be measured without any measurement error), an anomaly already pointed out by Gulliksen (1950).

Nevertheless, Equations (5) to (7) are technically correct. So how can the seemingly nonsensical behavior in Panel E be explained? The inconsistency comes from the fact that we assume that the variability in true scores across participants is equal to the variability in observed scores across participants and that we can therefore interpret the estimated true scores like observed scores (e.g., in Figure 1, as scores measured on a T-scale). However, we actually cannot do so, because compared to the standard deviation in observed scores *s_x_* (e.g., *s_x_* = 10 for observed scores that are standardized to *T*-scores as in Figure 1), the standard deviation in the underlying true scores *s_t_* decreases as the reliability decreases, following directly from the definition of reliability:

(8) $$r_{\mathrm{xx}}=\;\frac{s_{t}^{2}}{s_{x}^{2}}\;\to \;s_{t}^{2}=\;r_{\mathrm{xx}}\;\cdot \;\;\;s_{x}^{2}\;\to \;s_{t}=\;\sqrt{r_{\mathrm{xx}}}\;\cdot \;s_{x}$$

In the extreme case of zero reliability, the variance of the true scores across participants is even zero. Knowing this, it now makes perfect sense that the width of the confidence interval is zero when the reliability is zero. In this case, the regression-based true score estimate is equal to *M_x_* (e.g., 50 for *T*-scores) for all participants, regardless of their individually observed scores.

However, this is not just a funny anomaly for the admittedly unrealistic case of zero reliability, but has serious implications for the interpretation of test results across the whole range of reliabilities. As an example, consider a participant with an observed *T*-score of 70 (i.e., a score 2 *SD*s above the mean) and a test reliability of *r_xx_* = .50. Using Equation (5), the ETS for this participant is 60. A test administrator would now be tempted to interpret this score as being only 1 *SD* above the mean (because the T-scale has an *SD* of 10). However, this interpretation is incorrect because, as we know from Equation (8), the standard deviation of the true scores for a test with a reliability of .50 is not 10 but only $s_{t}=\;\sqrt{0.50}\;\cdot \;10=7.07$ . That is, the ETS of 60 is actually 1.4 *SD*, not 1 *SD*, above the mean of the true score distribution.

One possible solution could be for practitioners to take into account that the ETSs have a different metric than the observed *T*-scores when interpreting the test results. However, this is not easy to accomplish, as the metric depends on the reliability and the ETSs would therefore have to be interpreted differently for tests with different reliabilities.

## The Regression Approach With Scale Correction

So, what can be done to obtain true score estimates that are both unbiased and interpretable in the same way as norm-referenced observed values? The solution that I propose is simple: We need to rescale the true score estimates to preserve the scaling of the observed score. Only then can the ETSs be easily interpreted by practitioners independently of the reliability of the test. In the example above, the regression-based true score estimate of 60 (based on an observed *T*-score of 70) indicated a score that was 1.4 *SD* above the mean on the true score scale (i.e., it corresponded to a *z*-score of 1.4) because the standard deviation of true scores was actually $s_{t}=\;\sqrt{0.50}\;\cdot \;10=7.07$ and not 10, as one might wrongly expect. Converting the ETS of *z* = 1.4 to the standard T scale (*M* = 50, *SD* = 10) results in a rescaled estimated true score (RETS) of RETS = 50 + 10 · 1.4 = 64. The RETS then ends up on the same scale as the observed score and can be interpreted as a *T*-score.

Next, I provide equations that can be used to calculate the RETS and its standard error in one step (i.e., without the need to first estimate the ETS and then rescale this score in a second step, as I explained in the previous paragraph). To calculate the RETS, the ETS must be standardized using Equation (9) to obtain a score that is on the same scale as the observed score:

(9) $$\begin{array}{c} \mathrm{RETS}=\;M_{x}+\left(\frac{\mathrm{ETS}-\;M_{t}}{s_{t}}\right)\;\cdot \;s_{x}\\ \;\;=\;M_{x}+\left(\frac{M_{x}+r_{\mathrm{xx}}(x_{j}-M_{x})-\;M_{t}}{s_{t}}\right)\;\cdot \;s_{x} \end{array}$$

Because the mean of true scores *M_t_* equals the mean of the observed scores *M_x_* and because the standard deviation of true scores $s_{t}=\;\sqrt{r_{\mathrm{xx}}}\cdot \;s_{x}$ (see Equation (8)), it follows that:

(10) $$\begin{array}{c} \mathrm{RETS}=\;M_{x}+\left(\frac{M_{x}+r_{\mathrm{xx}}(x_{j}-M_{x})-\;M_{x}}{\sqrt{r_{\mathrm{xx}}}\;\cdot \;\;s_{x}}\right)\;\cdot \;s_{x}\\ \;\;=\;M_{x}+\;\sqrt{r_{\mathrm{xx}}}\;\cdot \;(x_{j}-M_{x}) \end{array}$$

During this rescaling, the standard deviation of the ETS was increased by the factor $\frac{s_{x}}{s_{t}}=\frac{s_{x}}{\sqrt{r_{\mathrm{xx}}}\;\cdot \;s_{x}}=\;\frac{1}{\sqrt{r_{\mathrm{xx}}}}$ . Accordingly, the standard error of the estimated true score (SEE) must be adjusted by the same factor to obtain the correct standard error for the RETS. The standard error of the rescaled estimate (SERE) is therefore given by:

(11) $$\begin{array}{c} \mathrm{SERE}=\;\frac{1}{\sqrt{r_{\mathrm{xx}}}}\;\;\cdot \;\mathrm{SEE}=\;\frac{1}{\sqrt{r_{\mathrm{xx}}}}\;\;\cdot \;s_{x}\;\cdot \;\sqrt{r_{\mathrm{xx}}}\;\cdot \;\sqrt{1-r_{\mathrm{xx}}}\\ \;\;=\;s_{x}\;\cdot \;\sqrt{1-r_{\mathrm{xx}}}=\mathrm{SEM} \end{array}$$

That is, the SERE is identical to the traditional SEM.

The confidence interval using the *regression approach with scale correction* can then be calculated as follows:

(12) CI=RETS±z·SEM

Panels G through I in the bottom row of Figure 1 show the confidence interval for the RETS, depending on the observed *T*-score and the test reliability. It can be seen that the RETS shows regression to the mean (i.e., the RETS moves closer to the mean as the reliability decreases). However, in contrast to the regression approach without scale correction shown in Panels D to F, the width of the confidence interval consistently decreases as the reliability increases, which is much more plausible from a diagnostic point of view, as more reliable information increases our certainty. And in the extreme case of zero reliability, the rescaled ETS is *T* = 50 with a 95% CI of 50 ± 1.96 · 10 = [30.4, 69.6]. This range is exactly what we would expect from a theoretical point of view because the observed test result provides no additional information at all due to its unreliability, but we know a priori that under the assumption of a normal distribution with a mean of 50 and an *SD* of 10, 95% of participants will have scores within the interval given above.

One may wonder why the estimated true scores are not just standardized using the observed standard deviation of the estimated true scores ( $s_{\mathrm{ETS}}=\;r_{\mathrm{tt}}\;\cdot \;s_{x}=0.50\;\cdot \;1=5$ ). However, such a procedure would not be expedient. If we standardized the estimated true scores using $s_{\mathrm{ETS}}$ , we would actually reverse the regression from Equation (5) and end up with the traditional confidence interval approach. Instead, for a correct interpretation of estimated true scores, we need to ensure that the estimated true scores are on the same scale as the observed scores, not that they have the same variance as observed scores. For this reason, we need to rescale the estimated true scores using the theoretical standard deviation of true scores from Equation (8), rather than restandardizing the estimated true scores using the observed standard deviation of the estimated true scores.^1^

## Simulation Studies

To further examine the proposed regression approach with scale correction, I conducted two simulation studies (the code can be found at https://osf.io/pfwav/). In the first simulation, responses to ordinal items with six ordinal categories were examined, which corresponds to typical questionnaires (e.g., personality tests) using Likert items. In the second simulation, responses to dichotomous items were examined, which corresponds to ability tests (e.g., *IQ* tests) in which an answer to an item is either correct or incorrect. In the first scenario, the assumption of CTT of a linear relationship between item responses and true values is reasonably fulfilled (at least as long as the distribution of item responses is approximately normally distributed as in the present simulation), whereas in the second scenario, the assumption of a linear relationship between item responses and true values is not reasonable and models from IRT are more suitable (e.g., using the logistic function).

In both simulations, normally distributed true scores on the standard T scale (*M* = 50, *SD* = 10) were simulated for 1,000,000 participants. In the first simulation, each of these participants’ responses to 10 items on a 6-point Likert scale were simulated. Item thresholds were chosen in a way to ensure approximately normally distributed item responses. In the second simulation, responses to 50 dichotomous items with varying item difficulty were simulated (cutoff thresholds were randomly drawn from a normal distribution with *M* = 50 and *SD* = 10). In both simulations, the amount of item error variance was varied to match different levels of reliability. Observed scores were then computed as the sum of the 10 ordinal items or 50 dichotomous items, respectively, and standardized to obtain observed scores that are T-scaled (*M* = 50, *SD* = 10). 95% CIs were estimated using all three approaches using Cronbach’s alpha as an estimate of the reliability. At last, I analyzed how well the 95% CIs estimated with the three approaches covered the actual true score, depending on different levels of reliability and depending on the observed *T*-score.

Table 1 presents the results of the simulation for a test consisting of ordinal items. As expected, the regression approach with scale correction resulted in confidence intervals that always covered about 95% of the actual true scores, regardless of the reliability of the test and regardless of the observed *T*-score. The other two approaches performed less well, and the lower the reliability, the worse they performed. Here, the regression approach without rescaling performed particularly poorly at low reliabilities, a consequence of the very small confidence interval for small reliabilities discussed earlier (see Figure 1, Panels D–F). In comparison, the traditional approach worked better and resulted in fairly accurate confidence intervals, especially for less extreme *T*-scores. This is because the traditional approach and the newly proposed approach for estimating the rescaled ETS both use the same SEM and differ only in whether or not regression to the mean is considered.

**Table 1:** Summary of the Simulation Study for Ordinal Items: Proportion of 95% CIs That Cover the Underlying True Scores.

Reliability	Traditional approach			Regression approach			Regression approach with scale correction		
	Observed *T* = 50	Observed *T* = 30 or 70	Across all *T*-scores	Observed *T* = 50	Observed *T* = 30 or 70	Across all *T*-scores	Observed *T* = 50	Observed *T* = 30 or 70	Across all *T*-scores
.10	.95	.70	.89	.47	.43	.46	.95	.95	.95
.20	.95	.77	.90	.62	.55	.60	.95	.95	.95
.30	.95	.81	.92	.72	.63	.70	.95	.95	.95
.40	.95	.84	.92	.79	.70	.77	.95	.95	.95
.50	.95	.87	.93	.84	.76	.82	.95	.95	.95
.60	.95	.89	.93	.87	.80	.86	.95	.94	.95
.70	.95	.91	.94	.90	.84	.89	.95	.94	.95
.80	.95	.92	.94	.92	.88	.91	.95	.94	.95
.90	.95	.94	.95	.94	.92	.93	.95	.95	.95

*Note. N* = 1,000,000 normally distributed true *T*-scores were simulated (*M* = 50, *SD* = 10). Then, for each true score, responses on 10 ordinal items with 6 categories were simulated based on the specified reliability. Observed scores were calculated as the sum of responses to the 10 items. 95% CIs were then calculated for each observed score using the three different statistical approaches. The proportions of confidence intervals that actually covered the underlying true score are presented separately for observed scores of *T* = 50, *T* = 30, and *T* = 70, as well as averaged across all simulated *T*-scores.

Table 2 shows the results of the simulation for a test consisting of dichotomous items. Results were generally comparable to those found for ordinal items, indicating that the regression approach with rescaling leads to confidence intervals with the best coverage. However, when reliability was high, coverage was less good for *T*-scores of 30 and 70. The reason for this is that we obtain more information for participants with an average true score because the item difficulties correspond better to their score (dichotomous items provide maximum information when the item difficulty corresponds to the participant’s true score). Consequently, the confidence interval for participants with an average true score is smaller than for participants with more extreme true scores. This dependence of the confidence interval on the true score can only be adequately captured if tests constructed using IRT are used. Here, a nonlinear relationship between the item response and the true score (e.g., a logistic function) is assumed instead of incorrectly assuming a linear relationship as in CTT.

**Table 2:** Summary of the Simulation Study for Dichotomous Items: Proportion of 95% CIs That Cover the Underlying True Scores.

Reliability	Traditional approach			Regression approach			Regression approach with scale correction		
	Observed *T* = 50	Observed *T* = 30 or 70	Across all *T*-scores	Observed *T* = 50	Observed *T* = 30 or 70	Across all *T*-scores	Observed *T* = 50	Observed *T* = 30 or 70	Across all *T*-scores
.10	.95	.72	.89	.47	.43	.46	.95	.95	.95
.20	.95	.76	.91	.62	.54	.60	.95	.95	.95
.30	.95	.82	.92	.72	.64	.70	.95	.95	.95
.40	.95	.85	.92	.79	.71	.77	.95	.95	.95
.50	.95	.87	.93	.83	.76	.82	.95	.95	.95
.60	.95	.89	.94	.87	.80	.86	.95	.95	.95
.70	.95	.91	.94	.90	.84	.89	.95	.94	.95
.80	.95	.92	.94	.92	.87	.91	.95	.94	.95
.90	.96	.89	.94	.95	.82	.93	.96	.87	.94

*Note. N* = 1,000,000 normally distributed true *T*-scores were simulated (*M* = 50, *SD* = 10). Then, for each true score, responses on 50 dichotomous items with randomly varying item difficulty were simulated based on the specified reliability. Observed scores were calculated as the sum of responses to the 50 items. 95% CIs were then calculated for each observed score using the three different statistical approaches. The proportions of confidence intervals that actually covered the underlying true score are presented separately for observed scores of *T* = 50, *T* = 30, and *T* = 70, as well as averaged across all simulated *T*-scores.

## Calculation of Accurate Confidence Intervals in Psychological Testing Practice

Confidence intervals that are based on the regression approach with scale correction can be calculated in the practice of psychological testing using Equations (10) to (12). Examples of calculations can be found in Box 1.

**Box 1:** Examples of the Calculation of Unbiased Confidence Intervals Using the Regression Approach With Scale Correction.

Ms. A has an observed *T*-score of 60 (*M_x_* = 50, *s_x_* = 10) on a personality test with a reliability of .80. What is the 95% CI for Ms. A’s true score? $\mathrm{RETS}=\;M_{x}+\;\sqrt{r_{\mathrm{xx}}}\;\cdot \;(x_{j}-M_{x})=\;50+\;\sqrt{0.80}\;\cdot \;(60-50)=\;58.94$ $\mathrm{SEM}=\;s_{x}\;\cdot \;\sqrt{1-r_{\mathrm{xx}}}=10\;\cdot \;\sqrt{1-0.80}=4.47$ 95%CI=RETS±z·SEM=58.94±1.96·4.47=[50.18,67.70] Mr. B has an observed *IQ* score of 90 (*M_x_* = 100, *s_x_* = 15) on an intelligence test with a reliability of .90. What is the 95% CI for Mr. B’s true score? $\mathrm{RETS}=\;M_{x}+\;\sqrt{r_{\mathrm{xx}}}\;\cdot \;(x_{j}-M_{x})=\;100+\;\sqrt{0.90}\;\cdot \;(90-100)=\;90.51$ $\mathrm{SEM}=\;s_{x}\;\cdot \;\sqrt{1-r_{\mathrm{xx}}}=15\;\cdot \;\sqrt{1-0.90}=4.74$ 95%CI=RETS±z·SEM=90.51±1.96·4.74=[81.22,99.80]

*Note*. SEM = standard error of measurement; CI = confidence interval; RETS = rescaled estimated true score.

However, to apply the regression-based ETS approach (regardless of whether with or without scale correction), one needs to know which group the person being tested comes from. This can be a difficult question that cannot always be answered unambiguously (see Nunnally & Kotsch, 1983, for a discussion on this topic; see also Hsu, 1995). Without further information about the participant, it is reasonable to assume that the participant comes from the total population and that the true score is regressed to the mean of the norming sample (e.g., *T* = 50). This is the case, for example, when a clinical symptom test is carried out for screening purposes on an unselected sample. However, if it is known a priori that a participant has been diagnosed with depression, then it is reasonable to assume that the true score is regressed to the mean of the clinical sample of individuals diagnosed with depression (e.g., *T* = 70). This means that in this case, the mean value used in Equation (10) should be the specific group mean value and not the mean value of the total population. In other cases, it may be unclear which group the participant belongs to, or the relevant statistics of the group may not be known, so it may be wisest not to take regression to mean into account and instead conservatively use the traditional confidence interval approach.

## Further Limitations of Confidence Intervals

When tests are based on the CTT, confidence intervals are identical across different true scores. This assumption can reasonably be questioned as the information obtained for a particular participant depends on the correspondence between the items administered and their score on the underlying dimension, which is most obvious for dichotomous items (i.e., for people with higher ability levels, difficult test items are more indicative of their ability, while people with lower ability levels are best measured with easy items). This dependence was also seen in the second simulation with dichotomous items reported in this manuscript, where all three different approaches for CTT tests underestimated the true confidence interval for more extreme test scores. Here, an accurate confidence interval would only be achieved using IRT instead of CTT, resulting in confidence intervals for a given test that are dependent on the participants’ true scores (Lord & Novick, 1968; Mellenbergh, 1996). However, this assumes that the corresponding test was constructed based on IRT so that the true scores and confidence intervals can be estimated based on IRT, which unfortunately is still often not the case.

Furthermore, all confidence intervals discussed so far in this article do not take into account the uncertainty in normed test scores due to sampling variability (Oosterhuis et al., 2017; Voncken et al., 2019). These limitations are, however, beyond the scope of this article, which aims to improve the calculation of confidence intervals in everyday testing practice for tests based on the CTT. However, when developing new tests, test authors should consider these limitations and the various solutions offered in the current psychometric literature for the best possible calculation of confidence intervals.

## Conclusion

Although the assumptions are not always fulfilled, most psychological tests used in everyday clinical testing practice are based on CTT. Therefore, it is of utmost importance for the practice of psychological assessment that the calculation of confidence intervals for tests constructed according to CTT is as accurate as possible. However, as has been repeatedly noted in the literature, the traditional approach to calculating confidence intervals within the CTT framework leads to biased estimates because it does not take into account the effect of regression to the mean.

For this reason, the regression-based approach for estimating true scores was introduced in the literature as a more accurate alternative for calculating confidence intervals. However, although the regression approach is technically correct, it results in true score confidence intervals that are on an arbitrary scale, making its results difficult to interpret. When the confidence interval calculated with this approach is misinterpreted as lying on the standard scale of the particular test (e.g., T scale, or IQ scale), which is likely to be the case in testing practice, then this approach, although theoretically better, leads to even more biased confidence intervals than the traditional approach. In this manuscript, a scale correction was introduced to solve the problems with the regression approach. As shown both analytically and in simulation studies, the regression approach with scale correction yields confidence intervals that are both unbiased and on the same scale as the observed test scores (i.e., this approach leads to confidence intervals that are on the IQ scale, the T scale, the Z scale, or any other scale used for standardizing the raw scores in the particular test).

The traditional approach, which is the easiest to calculate by hand and will be the most widely used in practice, does not fare so badly in the simulations. The reason is that the traditional approach and the rescaled ETS approach both use the same SEM and differ only in whether or not regression to the mean is considered. Therefore, the traditional approach only leads to inaccurate results when test scores deviate substantially from the mean. It thus seems reasonable to use the traditional approach, which is the easiest to calculate, in practice, at least for less extreme values and more reliable tests. However, the regression-based approach with rescaling should always be used when confidence intervals are calculated for printing in test manuals or when the test scoring is carried out with the aid of computers, as the more complex calculation is not an argument against its use here.

## Notes

1. This procedure is analogous to the standardization procedure in factor score models or IRT-based models. Here, the estimated true scores (factor scores, estimates of theta) are standardized using the model-based variance estimate of the true scores and not using the variance of the estimated true scores.
